# *Rosmarinus officinalis* L.: an update review of its phytochemistry and biological activity

**DOI:** 10.4155/fsoa-2017-0124

**Published:** 2018-02-01

**Authors:** Joana M Andrade, Célia Faustino, Catarina Garcia, Diogo Ladeiras, Catarina P Reis, Patrícia Rijo

**Affiliations:** 1 CBIOS – Research Center for Biosciences & Health technologies, Universidade Lusófona de Humanidades e Tecnologias, Campo Grande 376, 1749–024 Lisboa, Portugal; 2 iMed.ULisboa – Research Institute for Medicines, Faculdade de Farmácia da Universidade de Lisboa, Av. Prof. Gama Pinto, 1649–003 Lisboa, Portugal; 3 Biophysics & Biomedical Engineering Institute (IBEB), Faculdade de Ciências da Universidade de Lisboa, Campo Grande, 1749–016 Lisbon, Portugal

**Keywords:** biologic activity, carnosic acid, carnosol, essential oil, rosemary, rosmarinic acid, *Rosmarinus officinalis*

## Abstract

The worldwide interest in the use of medicinal plants has been growing, and its beneficial effects being rediscovered for the development of new drugs. Based on their vast ethnopharmacological applications, which inspired current research in drug discovery, natural products can provide new and important leads against various pharmacological targets. This work pioneers an extensive and an updated literature review on the current state of research on *Rosmarinus officinalis* L., elucidating which compounds and biological activities are the most relevant. Therefore, a search was made in the databases PubMed, ScienceDirect and Web of Science with the terms ‘rosemary’, ‘*Rosmarinus officinalis*’, ‘rosmarinic acid’ ‘carnosol’ and ‘carnosic acid’, which included 286 articles published since 1990 about rosemary's pharmacological activities and their isolated compounds. According to these references, there has been an increasing interest in the therapeutic properties of this plant, regarding carnosic acid, carnosol, rosmarinic acid and the essential oil. The present manuscript provides an updated review upon the most reported activities on *R. officinalis* and its active constituents.

## Lay abstract

The worldwide interest in the use of medicinal plants has been growing, and their beneficial effects being rediscovered for the development of new drugs. Actually, current research in drug discovery has been inspired on the vast ethnopharmacological applications of natural products, providing new and important leads against various pharmacological targets.

In this work, an updated literature review is presented to clarify the current state of research on *Rosmarinus officinalis* L., elucidating its constituents and their most relevant biological activities. Therefore, this work provides an updated review upon the most reported medicinal properties, namely, antitumoral, anti-inflammatory, analgesic, neurodegenerative, endocrinal, anti-infective and antioxidant.

Medicinal plants have been used worldwide by indigenous populations, playing an important role in the treatment of human and animal diseases [1]. More recently, the majority of modern drugs have been developed from isolated compounds of medicinal plants, based on their ethnopharmacological uses/applications [1–5].

The role of natural products on drug development has been increasing, not only when the bioactive compounds are directly used as therapeutic agents but also when they are used as raw material for drug synthesis, or as a base model for new biologically active compounds [6,7]. However, validating and using plants as a phytopharmaceutical requires a great deal of basic and applied research, in order to set this resource at the same level of importance of conventional pharmaceutical products [1].

Moreover, only about 10% of 250,000 species of plants estimated worldwide have been scientifically studied with potential use in healthcare [8,9]. Also, around 60,000 species will probably become extinct by the year of 2050, so it is urgent to search for new compounds with therapeutic interest [3].

*Rosmarinus officinalis* L. is a medicinal plant that belongs to the *Lamiaceae* family and is commonly known as rosemary [10]. Besides the culinary uses due to the characteristic aroma, this plant is also widely employed by indigenous populations, where it grows wild [4].

The extracts obtained from rosemary are used as a natural antioxidant, improving the shelf life of perishable foods [5]. In fact, the EU has approved the rosemary extract (E392) as a safe and effective natural antioxidant for food preservation [11].

Due to the growing interest on the medicinal properties of *R. officinalis* L., it is of great importance to review previous studies, establish professional links between the companies, government, the major pharmaceutical corporations and academic institutions [6,12–14]. Therefore, this manuscript builds a literature gathering on rosemary, an endemic species in Portugal to identify main bioactive compounds, extracts and essential oils and their connection with pharmacological activities. Also, it is intended to promote the development and expansion of this plant-based medicine (PBM), promoting a conscious exploitation of biodiversity as a source of chemical compounds and their use in the development of new lead drugs.

## Research method

A search was made with the databases PubMed, ScienceDirect and Web of Science for an update on rosemary pharmacological activities and main constituents. The search was performed using the terms ‘rosemary’, ‘*Rosmarinus officinalis*’, ‘rosmarinic acid’, ‘carnosol’ and/or ‘carnosic acid’, published since 1990.

## Inclusion & exclusion criteria

The search for this review was solely performed on English, Spanish and Portuguese languages since 1990, reporting pharmacological and chemical data, and some experimental studies on animals, using the isolated compounds, extracts and/or essential oils from *R. officinalis*. Finally, in order to grant reliability, only publications on peer-reviewed journals were chosen.

## Medicinal plants as a source of natural products

PBM has been employed throughout history and is nowadays the foundation of traditional medicine. Plants can be used as therapeutic resources in the form of herbal infusion, pharmaceutical preparations such as extracts, tablets or capsules by extracting and purifying active compounds [12,15]. Primitive communities in China, India and many other countries learned from experience [16] to distinguish the useful plants with beneficial effects, from those which were toxic or inactive, and to identify which combinations of plants were best for each illness [17].

Traditional medicine can be broadly classified into four basic systems: the Ayurvedic medicine (meaning ‘science of life’), which originated in India more than 5000 years ago; Chinese medicine, which is a part of traditional oriental medicine; African medicine; and Western medicine, originally from Greece and Rome, then spreading to Europe and to North and South America [17]. Ayurvedic medicine continues to be one of the oldest traditional medicine systems currently practiced in India, Sri Lanka and other countries, with over 1000 plants described in the Ayurvedic Pharmacopoeia [12,18]. There are several ancient records on the use of plants such as the *Ebers Papyrus* (1550 BC) and *De Materia Medica*, written by Dioscorides (77 AD). This last one describes over 600 medicinal plants, but during the Middle Ages, there was very little progress in the development of PBM [19].

Medicinal plants have an important role on pharmacological research and drug development, not only when the bioactive compounds are directly used as therapeutic agents, but also when they are used as raw material for drug synthesis or as a base model for new lead compounds [6,7]. However, validating this resource at the same level of conventional pharmaceutical products requires a great deal of basic and applied research [1].

A phytopharmaceutical preparation, or a PBM is any drug obtained exclusively from plants, in both raw and pharmaceutical formulation [15]. In many countries, PBMs are not regulated or controlled leading to poor quality control, consequently decreasing the acceptance and confidence in these products by the medical community [4,6–7].

In 1805, morphine became the first pure pharmacologically active compound to be isolated from a plant, although it was only in 1923 that its structure was elucidated [16]. Still in the 19th century, the first organic synthesis of urea, by Friedrich Wohler in 1828 [20], initiated the era of synthetic compounds. Even though synthetic drug development became the mainstay of conventional medicine [6], numerous alkaloids were isolated, such as atropine (*Atropa belladonna*), cocaine (*Erythroxylum coca*), ephedrine (species *Ephedra*), codeine (*Papaver somniferum*), pilocarpine (*Pilocarpus jaborandi* Holmes) and physostigmine (*Physostigma venenosum*), that are still widely used in drug prescription [16]. However, the interest in PBM for drug development was only restored in the early 1980s [6], due to the ineffectiveness of conventional medicine, namely: the cytotoxicity and side effects; the discomfort in the treatment of chronic diseases, in comparison with the use of phytopharmaceuticals; the abuse and misuse of synthetic drugs; the unavailable pharmacological treatments in a large percentage of the world population; and more importantly, the high costs involved in conventional medicine [2,4,11–13].

In 1997, the world market on medicinal products, not subject to medical prescription was 10 billion dollars, with an annual growth of 6.5% [12]. In 2001 and 2002, about a quarter of the top-selling drugs in the world were from natural products or their derivatives [21]. In 2003, the growth exceeded expectations, achieving sales of over 65 billion dollars, with 9 billion in Europe, with Germany as the European leader [12]. In fact, the use of the rosemary leaf has been approved by the German Commission for dyspepsia, high blood pressure and rheumatism at doses of 4–6 g/day, while the essential oil has been approved at doses of 0.1–1 ml [10]. In 2009, the total market value for PBM was about 83 billion dollars [9].

In accordance with the principles of phytotherapy, a plant contains a number of pharmacologically active compounds which must be seen as a single unit [22]. The entire extract can be standardized and clinically tested for a particular clinical condition. This feature differentiates phytotherapy from conventional pharmacotherapy [12]. Chemically speaking, natural compounds can lead to the active substances, not only allowing the planning and design of new drugs, but can also lead to the development of biomimetic synthesis (used as precursors) [23,24].

Although the industrial revolution and the development of organic chemistry have resulted in a preference for synthetic products, the WHO reports that in most of the developing countries about 80% of citizens still depend on the traditional medicine as main source of healthcare [25]. Nonetheless 25% of all prescribed drugs worldwide are derived from plants [17–18,26–27].

Presumably the production of phytotherapy drugs will require genetically uniform plants monocultures, grown in fully controlled conditions ensuring consistency and biochemical optimization of safety and efficacy of these products [28]. It is necessary to develop innovative technologies for isolation, purification and structural characterization for improved discovery and development of new PBMs [13,18,28].

## Medicinal plants as a source for drug development

Medicinal plants are a renewable source of compounds, providing an almost unlimited source of new and complex chemical structures [26,29]. Only some of these compounds were investigated or produced as synthetic drugs, such as vinblastine, vincristine, taxol and digoxin [26,29]. Examples of the discovery of new molecules from isolated compounds are irinotecan and topotecan (anticancer molecules), derived from an isolated compound from *Camptotheca* spp. and thereafter from *Mappia* spp. (camptothecin) [30].

Therefore, plants provide a desirable therapeutic effect with reduced risk of iatrogenic complications, such as side effects often associated with conventional medication [23]. The combined treatment of herbal medicines and synthetic drugs may reduce some adverse effects of highly potent drugs [23].

However, a major limitation on the use of plants in the phytopharmaceutical area is the lack consistency in the levels of compounds present in the extract due to natural variability, leading to inconsistent results upon scientific validation [31]. Also, a major limitation is the lack of reproducibility of the activity by more than 40%, when using plant extracts, as the activity detected often do not occur when the samples are re-extracted [27–28,32]. This problem is largely due to differences in the biochemical profiles of plants harvested at different times and locations, variations in the same genus plant and variations in the methods used for the extraction and determination of biological activity [28,32–33].

Drug development from medicinal plants faces yet another challenge. The bioactive compounds are generally isolated in small quantities, being insufficient for all stages of development and production of a new drug [16,18,21]. Therefore, the collaboration between researchers in different areas is essential to determine whether the total synthesis or semisynthesis is possible from the active compound.

Moreover, the effectiveness and activity of a phytochemical sample does not often result from the action of only one compound which is linked to the natural variability, already discussed, but from a synergic effect of several compounds (e.g., *Panax ginseng*) [28,32–33]. In most cases, this limitation leads to different results, which are difficult to interpret and to be accepted by the scientific community.

Regarding the lack of regulation, PBMs can be adulterated and contaminated in countries where purity and quality control are negligent [12,32]. In many cases, these adulterated products can cause significant health problems [32]. Ayurvedic products are often prepared with inorganic active compounds, which when combined with environmental pollution (such as pesticides), can increase the content of heavy metals above the permissible limits [32].

Standardization defined as the normalization of a sample that can have a minimal amount of one or more compounds is yet another setback [9]. Unfortunately, phytotherapy rarely satisfies the standardization norms [32,34]. This is mainly due to the lack of information about pharmacologically active compounds, and to the plants not being grown in a controlled environment [32,34]. The variability of the content and concentration of constituents in a plant, together with the various extraction and processing techniques used by different manufacturers, lead to variability in the content and quality of marketed PBM [23,29,32,34]. The consistency in composition and biological activity are prerequisites for the safe and effective use of therapeutic products [23,32].

## *Lamiaceae* family

Rosemary belongs to the *Lamiaceae* family, which is one of the largest and most distinguished families of flowering plants, including about 236 genera and 6900–7200 species worldwide [35,36]. The original family name is *Labiatae* because the flowers typically have petals fused into top and bottom lips, although currently most botanists use the name *Lamiaceae* [36].

The *Lamiaceae* is well known for its biologically active essential oils, common to many family members, its ornamental and culinary herbs such as basil, lavender, mint, rosemary, sage and thyme [35,37].

Several studies report the presence of a wide variety of compounds such as terpenes, iridoids, flavonoids and phenolic compounds in plants of the family. The *Lamiaceae* family includes species of plants containing large amounts of phenolic acids, such as rosmarinic acid, which have antibacterial, antiviral, antioxidant and anti-inflammatory properties [35].

Many species of this family have been the subject of experimental studies confirming the effectiveness of some of its traditional applications. *Thymus* spp. (thyme) has antibacterial activity due to the presence of thymol and can be used as a disinfectant; lavender oil, containing terpenic compounds, is used in the treatment of dandruff and hair growth, and also has antimicrobial, antiviral and antifungal properties; the aerial parts of *Stachys lavandulifolia* Vahl. are effective in the improvement of anxiety disorders due to the presence of apigenin and luteolin in the plant; *Lavandula angustifolia* Mill is used for inflammation, cough, as a sedative and in digestive problems; and compounds such as 1,8-cineole are very common in the genus *Nepeta*, with expectorant, antiseptic and antihelminthic activities [35].

The following sections will further address an important species of the *Lamiaceae* family, *R. officinalis* L. which is very common in Portugal, mainly focusing on its biological compounds and activities.

## *Rosmarinus officinalis Linnaeus*

The *R. officinalis* L. (rosemary) is one of the species in the genus *Rosmarinus* named by Carl Linnaeus (see scientific classification displayed in Table 1), and original from the temperate countries of the Mediterranean region, such as Portugal.

**Table 1.** **Scientific classification of *Rosmarinus officinalis* L.**

**Scientific classification**	
Kingdom	Plantae

Sub kingdom	Tracheobionta

Super division	Spermatophyta

Division	Magnoliophyta

Class	Magnoliopsida

Sub class	Asteridae

Order	Lamiales

Family	Lamiaceae

Genus	*Rosmarinus* L.

Species	*officinalis*

Binomial nomenclature	*Rosmarinus officinalis* L.

Data taken from [38].

Rosemary is a dense bush, branched, evergreen and blue–white flower, reaching a height of about 1 m [39,40]. It is characterized by leaves with 1–4 cm long and 2–4 mm wide, sessile, leathery, linear to linear-lanceolate, with curved edges, dark green upper side and granulosa and page bottom tomentous, with prominent midrib, and very characteristic smell [38,40–41].

**Figure 1.**
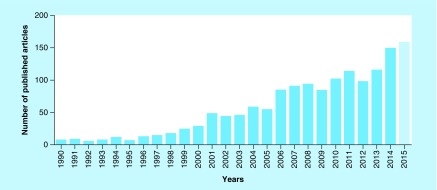
**Number of published papers in *Rosmarinus officinalis* L. since the 1990s until 30 November 2014, and the year of 2015, according to the databases searched in PubMed, ScienceDirect and Web of Science.**

In the past 20 years, there has been a clear tendency to rise in the number of articles regarding *R. officinalis* L. The interest in this plant is translated into the high amount of research performed since 2010, an average of 120 each year, a number that tends to increase, as shown in Figure 1.

In order to obtain the biologically active compounds from rosemary, it is necessary to obtain the plant's extracts and/or essential oils, and perform a phytochemical characterization. The extraction methods are applied to the plant most active portions (leaves, roots, stems or flowers), using selective solvents and standard procedures [42]. These techniques result in complex mixtures in liquid and semisolid forms or, after removal of the solvent, in the form of dry powder [43,44].

The qualitative and quantitative studies on bioactive compounds isolated from plants depend greatly on proper choice of extraction method, which plays a crucial role in obtaining satisfactory results (Table 2) [45]. The most important factors affecting the extraction process are related to the properties of the plant, the applied solvent, temperature, pressure and time of extraction [43–45]. There are classical extraction methods such as Soxhlet extraction, maceration, decoction and infusion, and modern methods, such as supercritical fluid extraction and solid-phase micro-extraction, among others [44,45].

**Table 2.** **Some examples of bioactive compounds that can be extracted with different solvents.**

**Water**	**Ethanol**	**Methanol**	**Chloroform**	**Dichloromethane**	**Ether**	**Acetone**
Anthocyanin Tannin Saponin Terpenoid	Tannin Polyphenol Flavonol Terpenoid Alkaloid	Anthocyanin Terpenoid Saponin Tannin Flavone Polyphenol	Terpenoid Flavonoid	Terpenoid	Alkaloid Terpenoid	Flavonoid

Water	Ethanol	Methanol	Chloroform	Dichloro methane	Ether	Acetone

Data taken from [45].

After analysis of the collected articles, the most used extraction methods to obtain the bioactive compounds from *R. officinalis* (see tables in Supplementary material) are maceration, hydrodistillation, distillation and Soxhlet by supercritical fluid extraction.

The essential oils are complex mixtures that contain hundreds of compounds, volatiles, monoterpenes, sesquiterpenes, aromatic compounds and other derivatives [46]. The essential oil of rosemary obtained by steam distillation from the leaves (up to 2.5%) is colorless to light yellow, water-insoluble and with a characteristic aroma of camphor [38–39,41,47]. The main constituents of the rosemary essential oil are camphor (5.0–21%), 1,8-cineole (15–55%), α-pinene (9.0–26%), borneol (1.5–5.0%), camphene (2.5–12%), β-pinene (2.0–9.0%) and limonene (1.5–5.0%) in proportions that vary according to the vegetative stage and bioclimatic conditions [38,40].

Regarding the extracts, the phytochemicals mainly present in *R. officinalis* are rosmarinic acid, camphor, caffeic acid, ursolic acid, betulinic acid, carnosic acid and carnosol [19,38,40,48]. Therefore, *R. officinalis* is mainly composed of phenolic compounds, di- and triterpenes and essential oils [49,50].

In traditional medicine, the leaves of *R. officinalis* L. are used based on their antibacterial activities [51], carminative [9,38,52] and as analgesic in muscles and joints [38–40,53]. Also, rosemary's essential oils and extracts obtained from flowers and leaves are used to treat minor wounds, rashes, headache, dyspepsia, circulation problems, but also as an expectorant, diuretic and antispasmodic in renal colic [38–40,48].

Polyphenols are antioxidant chemical compounds primarily responsible for the fruit coloring, which are classified as phenolic acids, flavonoids and nonflavonoids [54]. In addition to their antioxidant properties, they play a very important role in the plant defenses against herbivores, pathogens and predators; therefore, they have an application in the control of infectious agents in humans [54]. In *R. officinalis*, the most common polyphenols are apigenin, diosmin, luteolin, *genkwanina* and phenolic acids (>3%), especially rosmarinic acid, chlorogenic acid and caffeic acid [19,37,39–40].

**Figure 2.**
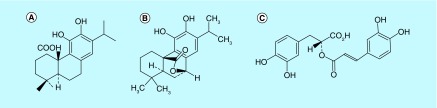
**Chemical structure of three major compounds present in *R. officinalis*.** **(A)** Carnosic acid. **(B)** Carnosol. **(C)** Rosmarinic acid.

Other major compounds common in rosemary are terpenes, usually present in essential oils and resins, which include over 10,000 compounds divided into mono-, di-, tri- and sesquiterpenes, depending on the number of carbon atoms and isoprene groups (C_5_H_8_) [46,54]. It is possible to find in rosemary terpenes such as epirosmanol, carnosol, carnosic acid (tricyclic diterpenes: see structure in Figure 2), ursolic acid and oleanolic acid (triterpenes) [19,38,40,48].

However, the carnosic acid, which is converted to carnosol by oxidation, has physicochemical, thermal and photolabile properties, which can be avoided by a supercritical fluid extraction (low temperature operation) [55].

In 2014, five new compounds were identified in an ethanolic extract of *R. officinalis*, the officinoterpenoside A_1_ and A_2_ (diterpenoid glycosides), officinoterpenoside B and C (triterpenoid glycosides) and officinoterpenoside D (normonoterpenoid) [56].

Regarding the most studied compounds from *R. officinalis* and their biological activities, the increased pharmacological potential is clear for carnosic acid and the essential oil of rosemary. This issue will be further reviewed in the next section.

## Biological activities of *R. officinalis* compounds

Rosemary has been widely used not only in cooking, especially to modify and enhance flavors, but also in traditional medicine, being a highly appreciated medicinal plant to prevent and cure colds, rheumatism, pain of muscles and joints [56,57]. It is nowadays one of the most popular sources of natural bioactive compounds, and in fact, this plant exerts various pharmacological activities such as antibacterial [51], antidiabetic [58], anti-inflammatory [59,60], antitumor [61–63] and antioxidant [64], among others [56].

After revising and analyzing the items of the gathered bibliographic data, the most studied compounds from *R. officinalis* were identified.

**Figure 3.**
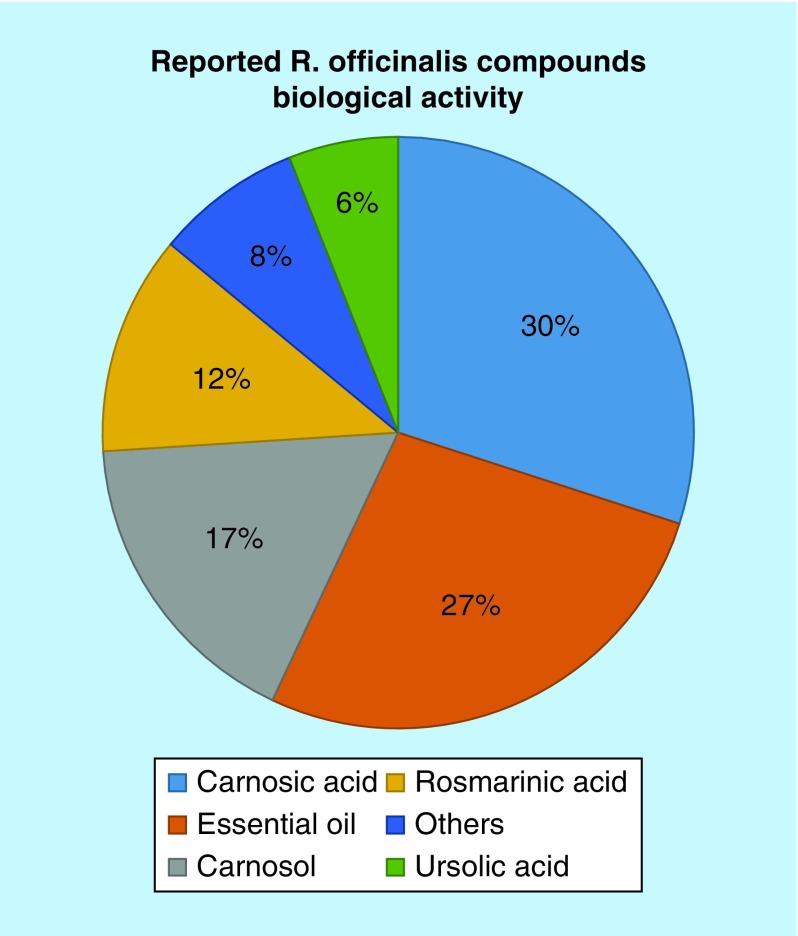
**Percentage of biological activity studies associated with each compound, from all investigated articles.** Studies on the carnosic acid include the compounds 12-*O*-metilcarnosic acid and 12-methoxy-cis/trans-carnosic acid. Studies on the essential oil include the compounds 1,8-cineol, α-pinene and β-pinene.

The graph of Figure 3 shows that carnosic acid is the most investigated bioactive compound with about 30% of the studies, together with the essential oil, followed by carnosol, rosmarinic acid and ursolic acid, which comprised 35% of the research studies.

Compounds such as betulinic acid, oleanolic and micromeric, rosmanol, epirosmanol and luteolin, among others, have not been the target of extensive studies, corresponding only to 23 studies out of 275. However, there has been an increasing interest in these compounds since 2010 (Table 3).

**Table 3.** **Evolution of studies with compounds over the years according to all reviewed articles.**

	**Carnosol**	**CA**	**RA**	**UA**	**EO**	**Others**	**Total**
1990	0	0	0	0	0	0	0

1991	0	0	0	0	1	0	1

1992	1	2	0	0	0	0	3

1993	0	0	0	0	2	0	2

1994	2	0	0	2	0	0	4

1995	2	1	0	0	0	0	3

1996	2	0	0	0	0	0	2

1997	0	0	0	0	0	0	0

1998	0	0	0	0	0	0	0

1999	0	0	0	0	4	0	4

2000	0	0	0	0	1	0	1

2001	2	2	0	0	1	2	7

2002	2	4	2	3	0	0	11

2003	2	3	1	0	1	1	8

2004	1	3	0	0	2	1	7

2005	2	0	0	0	0	0	2

2006	3	5	2	0	3	0	13

2007	1	4	0	1	8	2	16

2008	1	5	1	0	4	0	11

2009	0	2	2	1	1	1	7

2010	7	10	5	2	12	3	39

2011	3	9	2	2	11	2	29

2012	5	10	5	3	8	4	35

2013	8	10	11	1	5	7	42

2014	2	14	1	1	10	0	28

CA: Carnosic acid; EO: Essential oil; RA: Rosmarinic acid; UA: Ursolic acid.

There is clearly, according to the scientific community, a great pharmacological potential in *R. officinalis*, especially with carnosic acid and the essential oil, which is apparent from the increasing number of studies in the last 5 years, with 53 and 46 studies, respectively.

The biological activities of the *R. officinalis* L. have been attributed to two groups of compounds: a volatile fraction and phenolic compounds [65]. This last group mainly contains a fraction of flavonoids, rosmarinic acid and some diterpene compounds structurally derived from carnosic acid, carnosol and rosmanol [65].

**Figure 4.**
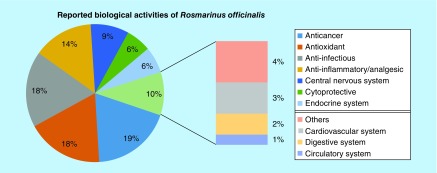
**Ratio on the number of studies (%) to the studied biological activities on *R. officinalis* according to the literature.**

From the articles reviewed, it was eminent that in the last 20 years, rosemary has been most studied considering its anticancer, antioxidant and anti-infectious properties, encompassing 55% of the studies. The activities related to the CNS (antidepressant, neuroprotective, cholinergic, etc.), anti-inflammatory and analgesic effects accounted for almost 25% of the studies (Figure 4).

## Antitumor activity

The composition of the human diet can influence the risk of cancer and its components can exert positive or negative influences [66]. Chemoprevention is the long-term pharmacological control on the risk of cancer. On this matter, several plants, together with their compounds, have been investigated for their antitumor potential [66,67]. About 70% of the drugs used in cancer treatment derive from natural products [68].

As previously described, rosemary is known to exert antioxidant activity thus inhibiting genotoxicity, and protecting from carcinogens or toxic agents [69]. However, the pronounced side effects of the therapeutic methods largely prevent its effectiveness, increasing the demand for new approaches in cancer treatment and prevention [70].

Polyphenols are compounds capable of modulating cell growth and differentiation and thus interfere with tumor development and progression [71]. Since rosemary is rich in phenolic compounds, many studies have been targeted for antitumor activity (about 20%) (Figure 4) [62,72–75].

Carnosic acid and carnosol are diterpenes that represent about 5% of *R. officinalis* dried leaves weight, and these compounds have greater antitumor relevance (35 in 49 studies use these compounds). There has been a large increase in the number of studies regarding the antitumor activity of carnosic acid, carnosol, rosmarinic acid, ursolic acid over the past 5 years (Table 3). Breast cancers, melanoma, colon cancer, liver carcinoma and leukemia have been the most studied. In fact, there has been several *in vitro* studies regarding cytotoxicity of carnosol and carnosic acid to human cancer cells (HepG2, COLO 205 and HL-60), breast cancer cells and colon cancer cells [73,76]. These studies reported a decrease of cell viability using carnosic acid in a dose-dependent manner, including resistant tumor cells, suggesting this compound as a complementary antitumoral approach [77].

From *in vivo* experiments, carnosic acid and its ester derivatives were found to be effective in preventing gastric lesions in HCl/EtOH-induced gastric lesions model in mice [78]. Also, treatment with rosemary extract of 7,12-dimethylbenz[a]anthracene (DMBA)-induced mammary tumorigenesis in rats, suggested a chemopreventative action for experimental mammary tumorigenesis [79].

More detailed information concerning *in vitro* and *in vivo* experimental studies is displayed in the tables in Supplementary material.

## Antioxidant activity

The natural antioxidants from plants are becoming increasingly important, not only in the nutritional area (food preservation and stability) but also in preventive medicine [80]. The *Lamiaceae* family has been a focus of the research on antioxidant compounds due to its high polyphenol content [81]. Likewise *R. officinalis* leaves are commonly used as a condiment for flavoring food, and as a source of antioxidant compounds employed in food conservation [82].

Antioxidants play a major role in the prevention and treatment of diseases associated with oxidative damage, including cancer, cardiovascular and neurodegenerative diseases [80,83–84]. Reactive oxygen species, including hydrogen peroxide and free radicals, such as superoxide anion (O_2_^•-^) and hydroxyl radical (HO^•^), are inevitably produced in living organisms resulting from metabolic processes or from external sources [81]. Continued exposure to free radicals in biological systems may cause functional and structural damage, aging and cell death [85].

Several *in vitro* studies were reviewed regarding the antioxidant activity of the main isolated compounds from rosemary, namely, carnosol, carnosic acid, rosmanol, rosmarinic acid, oleanolic acid and ursolic acid. Using the 2,2-diphenyl-1-picrylhydrazyl method, these bioactive compounds and the essential oil were validated for their antioxidant activity [86–88]. Also, using the thiobarbituric acid, superoxide anion and lipid free radicals scavenging activity assays and Rancimat methods (determination of oxidative stability of fat), the bioactive compounds carnosol, rosmanol and epirosmanol have been reported to inhibit lipid peroxidation through the lipid free radical scavenging mechanism [58,89–90]. These studies have shown the antioxidant potential of rosemary phytochemicals, whose properties are closely related to other biological activities, such as cytoprotective and anticancer, primarily due to their ability to neutralize reactive oxygen species.

Considering *in vivo* studies, there were only three different studies found until 2014 for the validation of *in vitro* experimental results, using the essential oil and carnosic acid. These studies were performed using Wistar rats, and evaluated the catalase, glutathione peroxidase, superoxide dismutase and nitric oxide synthase activities, as well as lipid peroxidation and reactive oxygen species, in brain and heart tissues after diet supplementation with rosemary essential oil. This resulted in the decrease of oxidative stress, since dietary rosemary has the potential to quench free radicals, inhibit lipid peroxidation and improve the antioxidant status in rat tissues [55,81,91].

More detailed information concerning *in vitro* and *in vivo* experimental studies is displayed in the tables in Supplementary material.

## Anti-infectious activity

Most plants produce antimicrobial secondary metabolites, either from its normal course of growth and development, or in response to stress or pathogen attack. The use of essential oils represents a new way to reduce the proliferation of microorganisms [92]. *Rosmarinus officinalis* L. is widely used today as a food preservative and known for its powerful antibacterial activity [93].

The increasing use of antibiotics in medicine, agriculture and livestock has largely contributed to the increase of multiple drug resistant microorganisms [94]. Antimicrobial resistance is a global public health concern, and researchers have been increasingly engaging in this area in demand for new effective antimicrobial bioactives [94,95].

Besides the antibacterial properties, essential oils also have insecticidal, antiparasitic and antifungal activities, which are important for the control of human diseases of microbial origin [96].

Since the 1990s until 2014, the essential oil of rosemary has demonstrated the highest antimicrobial activity, with 65% of anti-infectious activity studies. The antimicrobial activity of the essential oil was superior, when compared with the single compounds 1,8-cineole and α-pinene [97].

Experimental *in vitro* studies, concerning MICs, minimal bactericidal concentration and time-kill dynamic processes, have reported that there is a possible synergistic effect between the antimicrobial compounds in essential oil [96,98]. These studies were performed testing carnosic acid, carnosol, rosmarinic acid, oleanolic acid, ursolic acid and essential oil, against Gram-positive bacteria (*Staphylococcus epidermidis*, *Staphylococcus aureus* and *Bacillus subtilis*), three Gram-negative bacteria (*Proteus vulgaris*, *Pseudomonas aeruginosa* and *Escherichia coli*) and two fungi (*Candida albicans* and *Aspergillus niger*). All reported pronounced antibacterial and antifungal activity (20; 227; 87; 136; 97; 93). It was also found that carnosic acid showed antiviral activity against human respiratory syncytial virus [99].

More detailed information concerning *in vitro* experimental studies is displayed in the tables in Supplementary material.

## Anti-inflammatory & analgesic activities

Plants are a good source of anti-inflammatory agents and the continuous search for new compounds, especially from plants with historically documented pharmacological effects, represents a huge pharmaceutical potential [82].

Controlling the release of mediators in the inflammatory process is the main objective of anti-inflammatory drugs [100]. Pain and inflammation are related to wound healing and production of free radicals which could extend the inflammation process [101]. Thus, the inflammatory response and the oxidative damage are two main factors that induce cardiovascular and neurodegenerative diseases; however, polyphenols from some plants are able to reduce these problems [102].

In folk medicine, rosemary is known for its therapeutic properties against abdominal pain and for the treatment of respiratory inflammatory diseases, such as bronchial asthma [103].

Some experimental studies have reported the anti-inflammatory and analgesic activities of the essential oil and biologically active terpenes such as carnosic acid, carnosol, ursolic acid and betulinic acid, as well as rosmarinic acid, rosmanol and oleanolic acid [82]. In fact, it has been reported that they have antinociceptive activity, and that each individual triterpene showed a similar potency to that observed with ketorolac, a nonsteroidal anti-inflammatory drug [82,104].

Regarding *in vitro* studies, the anti-inflammatory and analgesic studies were based on the evaluation of the expression of inflammatory cytokines (IL-1β, IL-6, TNF-α, etc.), COX-1/COX-2, iNOS and evaluation of nitric oxide production in RAW 264.7 macrophages cells [72,86,105–106]. Also, studies concerning the antiatherosclerotic effects of rosemary have been developed, through the migration and matrix metalloproteinase activation of vascular smooth muscle cells. These studies reported that carnosic acid has an ability to suppress matrix metalloproteinase-9 expression through downregulation of NF-κB and therefore decrease the smooth muscle cells migration [107,108].

It is apparent that the anti-inflammatory and analgesic activities have been the most studied, and there is an attempt to explain these properties with several compounds of *R. officinalis*, with carnosol the most investigated (Figure 4). These *in vitro* studies have shown a potential application for the prevention of inflammatory diseases [106].

At preclinical stages, the essential oil of rosemary has been used topically for muscular and rheumatic pains, evaluated using carrageenan-induced pleurisy and carrageenan-induced paw edema tests in rats. These studies suggested that the essential oil could remarkably decrease the induced edema in 1–4 h and significantly reduce the volume of pleural exudate, being both anti-inflammatory and antinociceptive [60,109]. Also, new lead compounds obtained by rosmarinic acid derivatization have been tested for these activities. Particularly, the acetyl derivative can reduce the induced paw edema as well as the paw licks, suggesting a potential application of this compound as anti-inflammatory and antinociceptive agent [109]. Regarding clinical trials in this topic, there have been several studies since 2005. Lukaczer *et al*. reported the efficacy of ‘Meta050’ (a combination of reduced iso-alpha-acids from hops, rosemary extract and oleanolic acid), in patients with rheumatic disease. Patients with pain caused by osteoarthritis, rheumatoid arthritis and fibromyalgia were given 440 mg of ‘Meta050’, which doubled after 4 weeks in the majority of patients. This work reports a significant decrease in pain in arthritis subjects by 40–50%, but not for fibromyalgia subject scores [110].

The *ex vivo* clinical study performed by Minich *et al*. on pain relief used a combination similar to ‘Meta050’, but is here presented as NG440. The goal was to examine the clinical safety and efficacy of NG440. The report suggested that NG440 is safe for human consumption and that animal toxicity revealed no adverse effects, at daily dosages ≤250 mg/kg. According to these results, NG440 may serve as an alternative where specific COX-2 inhibitors have been traditionally used [111].

Other work performed by Rosenbaum *et al*. [105] reported the use of triterpenes, ursolic acid, oleanolic acid and micromeric acid as dietary supplements for osteoarthritis and rheumatoid arthritis. These compounds (obtained from different extracts of rosemary leaf), were able to decrease topical anti-inflammatory activity, which was tested using the croton oil ear test in mice.

However, it is not clear if any of these supplements can be effectively and safely recommended to reduce nonsteroidal anti-inflammatory drug or steroid usage, thus, more research is required. More information is displayed in the tables in Supplementary material.

## CNS & endocrine system activities

Diabetes mellitus is one of the most prevalent metabolic disorders in the world. Insulin and oral hypoglycemic agents are used to treat diabetes; however, these drugs do not cure the disease and have significant adverse effects [112]. Rosemary has been shown to decrease blood glucose in several *in vivo* studies [113].

Some *in vitro* and *in vivo* studies have reported an inhibition of gastric lipase in the stomach of Zucker rats, after treatment with high contents of carnosic acid, which resulted in an improve of the triglycerides profiles [65,113]. Therefore, carnosic acid together with carnosol, proved to be the most relevant compounds on glycemic control.

Furthermore, there has been a growing trend of studies for new neuroprotective drugs from natural sources, which raises a new therapeutic hope [114].

Diseases of the CNS (depression, Parkinson, Alzheimer, etc.) are incurable chronic conditions, and presumably that is why there has been an increasing number of studies in *R. officinalis* over recent years, in an attempt to find new solutions. Regarding depression there are several studies reporting a decreasing immobility time and regulation of several neurotransmitters (dopamine, norepinephrine, serotonin and acetylcholine) and gene expression in mice brain like TH, PC and MAPK phosphatase (MKP-1) [115,116]. These studies contribute to the understanding of molecular mechanism behind the antidepressant effect of *R. officinalis* and its major active compounds.

Rosmarinic acid, however, seems to have potential against neurodegenerative diseases. It was found that this compound had cholinergic and neuroprotective effects and inhibited acetylcholinesterase [114,117].

Regarding clinical trials, in 2010, Pengelly *et al*. performed a randomized, placebo-controlled, double-blinded, repeated-measures crossover study to investigate possible acute effects of dried rosemary leaf powder on cognitive performance. This work reported significant speed of memory – a potentially useful predictor of cognitive function during aging, using rosemary powder at the dose normally used at culinary consumption. This work expands the value of future studies on effects of low doses of rosemary on memory and cognition [118].

More detailed information concerning *in vitro* and *in vivo* experimental studies is displayed in the tables in Supplementary material.

## Discussion

A total of 286 articles were obtained from the searched databases, comprising 232 based on biological activities (knowing that some publications report more than one activity) and 275 based on biological activity regarding isolated compounds. Publications were excluded based on restricted access to abstract, research area on nutrition (food supplements or stability) and irrelevant or unreliable results on biological activities. This review allows us to connect the biological activities of *R. officinalis* isolated compounds under search with essential oils and/or extracts.

From this review, it can be concluded that *R. officinalis* has a promising future especially in the treatment and prevention of various diseases, such as cancers, infectious diseases and CNS disorders. Interestingly, using the database from ClinicalTrials.gov with the same keywords under study, we found two clinical trials for rosmarinic acid and oleanolic acid, three studies for ursolic acid and none for carnosic acid or carnosol. These studies concern the topics of osteoarthritis of the knee, nasal polyps, GI tract, cardiovascular diseases, metabolic syndrome X, sarcopenia and benign prostatic hyperplasia. Thus, it is imperative to improve clinical studies of this medicinal plant, and learn from the traditional health practitioners that bear the knowledge of many generations of trial and error. The worldwide interest in the use of rosemary has been growing with its beneficial effects being rediscovered for the possible development of new drugs. Therefore, the need for new therapeutic agents with specific targets and less adverse side effects, supports the need to extend both the clinical and preclinical studies on *R. officinalis* plants.

## Conclusion

In recent years, much effort has been devoted to the development of PBMs [22], proposing them as natural drugs in many pathological conditions including anti-inflammatory, analgesic, antioxidant, antitumor, anti-infectious, CNS and endocrine system activities.

Phytotherapy is a major contributor to the discovery of new, safer and effective PBMs, as well as new drugs, knowing that what pharmaceutical chemistry has sought desperately, nature has in large quantities [12].

Based on the development of high-accuracy analytical methods and the advances of molecular biology and genetics, it is now possible to isolate plant compounds that exist in extremely small quantities [31]. With these improvements, it is now possible to study their chemical structure and therapeutic potential, and thus, modify the molecule for producing new, more selective therapeutic agents [12,31,119].

This work performed an updated review on *R. officinalis* L., allowing us to emphasize the current state of the art, on studies and investigations, pointing out the awareness of the pharmacological activities related to the constituents of this plant. Since 1990s, there has been an increasing therapeutic interest in rosemary, from both *in vitro* and *in vivo* studies on several biological activities, such as antioxidant, anti-inflammatory, analgesic, etc., previously described in ethnobotanicals studies.

From the reviewed literature, it can be concluded that the most important constituents of *R. officinalis* L., which are pharmacologically active and the main target of scientific studies, are carnosic acid, carnosol, rosmarinic acid and the essential oil. Thus, these natural drugs can be proposed for preclinical and clinical studies in different diseases and pathological conditions.

*R. officinalis* has a promising future in the medical field, especially in the treatment and prevention of various cancers, infectious diseases and increasingly emerging diseases such as depression, Alzheimer's and Parkinson's diseases. In fact, there are 80 clinical studies on *R. officinalis*, from which 32 are still open studies.

These studies regarding herbal remedies should be taken into more consideration since the safety and efficacy of many herbal medicines are still problematic, with inadequate or inconsistent methods. Considering this, more reliable trials are needed in the future to evaluate the *R. officinalis* active phytocompounds safety and efficacy, in treating different pathological conditions.
